# Polymorphisms of serotonin transporter gene and psychological status in patients with multiple sclerosis

**Published:** 2018-07-06

**Authors:** Shirin Farjadian, Bahareh Fakhraei, Zahra Niknam, Mahboubeh Nasiri, Aslan Azad, Mojtaba Farjam, Alireza Nikseresht

**Affiliations:** 1 Department of Immunology, School of Medicine, Shiraz University of Medical Sciences, Shiraz, Iran; 2 Department of Psychiatry, School of Medicine, Shiraz University of Medical Sciences, Shiraz, Iran; 3 Iran Neurological Association, Tehran, Iran; 4 Allergy Research Center, Department of Immunology, Shiraz University of Medical Sciences, Shiraz, Iran; 5 School of Pharmacy, International Branch, Shiraz University of Medical Sciences, Shiraz, Iran; 6 Noncommunicable Diseases Research Center, School of Medicine, Fasa University of Medical Sciences, Fasa, Iran; 7 Department of Neurology, School of Medicine, Shiraz University of Medical Sciences, Shiraz, Iran

**Keywords:** Multiple Sclerosis, Depression, Serotonin Reuptake Transporter Protein, C. Elegans, Genetic Variation

## Abstract

**Background:** Multiple sclerosis (MS) is the most common neuroinflammatory disease in young adults. Anxiety and depression may predispose individuals to MS and flare-ups. Serotonin transmission is modified in some brain regions of patients with MS, and these changes may contribute to their psychiatric abnormalities. We studied the frequencies of common polymorphisms of the serotonin reuptake transporter (SERT) gene in patients with MS according to their psychological status.

**Methods:** The 5-HTTLPR, rs25531, and STin2VNTR polymorphisms of the SERT gene were genotyped by polymerase chain reaction (PCR)-based methods in 100 patients with MS and 100 healthy controls.

**Results:** There were no remarkable differences in SERT gene polymorphisms between patients with MS and healthy controls. Unlike the control group, 41% of the patients showed some degree of depression based on Beck Depression Inventory (BDI), but no association was observed between SERT gene polymorphisms after the patients were stratified by depression status.

**Conclusion:** In addition to SERT gene polymorphisms, modulation of serotonin at the synapses may also be regulated by genetic variations in tryptophan hydroxylase type 2 and serotonin receptors. Further studies with functional brain imaging of the serotonergic system in patients with MS can provide information on the role of serotonin in this disease.

## Introduction

Multiple sclerosis (MS) is the most common neuroinflammatory disease that causes neurological deficits in young and middle-aged adults.^1^^,^^2^ Both genetic and environmental factors are seemed to be involved in the etiology of MS. In addition to certain human leukocyte antigen (HLA) alleles, genetic variation of interleukin-2 receptor alpha chain (IL2RA), IL7RA, kinesin family member 1B (KIF1B), H6PD, and tumor necrosis factor receptor superfamily-1 (TNFRSF1) are reported to be associated with MS.^3^

Some patients experience progressive devastating disease, and many others have a relapsing-remitting course that can lead to wide-spectrum chronic disabilities^4^ including cognitive deficits^5^ and psychiatric problems.^6^ Psychiatric disorders as anxiety and depression are common comorbidities among the patients with MS.^7^ These comorbidities adversely affect the course of MS.^8^ On the other hand, anxiety and depression is commonly believed to predispose individuals to MS or flare-ups of the disease. Although the correlation between MS and these disorders has been found, the pathophysiologic basis for this correlation has not been elucidated. Moreover, the optimal pharmacotherapeutic options for neuropsychiatric comorbidities in patients with MS are debatable, although a significant number of patients need to be treated with psychiatric medication.^9^

Serotonin (5-hydroxytryptamine) plays a pivotal role in the pathophysiology of most neuropsychiatric disorders.^10^ Serotonin level itself is controlled by serotonin reuptake transporter (SERT) gene on chromosome 17q11.2, and different polymorphisms affect the transcription activity of this gene, leading to impaired serotonin reuptake. A 44-bp insertion/deletion in the SERT gene promoter region with long (L) and short (S) forms affects gene transcription activity. The S allele is dominant to the L allele, whereas the L allele induces a two- to threefold higher level of SERT gene transcription than the S allele. The L/L genotype is associated with higher levels of SERT gene products than S/S or L/S.^11^ There is a single nucleotide polymorphism in the L allele, rs25531 (A to G), and the L_G_ allele is associated with decreased expression of the SERT gene.^12^ STin2VNTR, a 17-bp variable number of repeat in the second intron, is another polymorphic site in the SERT gene that results in alleles carrying 9-, 10-, or 12-repeat units. Transcriptional activity of the SERT gene is also affected by polymorphism at this site, as alleles with 9 or 12 repeats are associated with higher transcriptional activity than the 10-repeat allele.^13^

The changes and roles of this neurotransmitter in MS has been studied. Knocking out the SERT gene in experimental autoimmune encephalomyelitis (EAE), an animal model of MS, had some effect on inflammatory cell infiltration in the central nervous system (CNS).^14^ Serotonin transmission has been shown to change in some brain regions including the limbic, paralimbic, and frontal cortex in patients with MS, and this may contribute to psychiatric abnormalities in these patients.^15^^,^^16^ Specific serotonin reuptake inhibitors decreased the severity of neurological deficits in EAE, and had a neuroimmunomodulatory function.^17^ In clinical studies, the beneficial effects of selective serotonin reuptake inhibitors (SSRIs) have been reported as a reduction in relapses and radiological improvements in patients with MS.^18^ Relapses induced by stress were decreased by the administration of escitalopram, an SSRI.^19^^,^^20^ In addition, the high prevalence of neuropsychiatric comorbidities and the suspected role of serotonin and SERT in these diseases highlight the possible involvement of serotonin in the pathogenesis of MS.^20^

To evaluate the effect of serotonin on MS and neuropsychiatric comorbidities, we studied common polymorphisms of the SERT gene in patients with MS according to their psychological status, and compared the findings to those in healthy individuals.

## Materials and Methods

One hundred patients from southwestern Iran with proven MS based on the McDonald criteria^21^ were included in this study. A total of 100 unrelated sex- and age-matched healthy individuals from the same geographical region were included as the control group. The study protocol was approved by our University Ethics Committee.

After each participant had provided his/her informed consent in writing, all participants were asked to complete Beck Depression Inventory (BDI-II) to evaluate their depression status. The total score on this questionnaire (which consists of 21 multiple-choice items) was calculated by adding the scores for each answer, which ranged from 0 to 3. Based on the commonly accepted cut-off scores for grading the severity of depression, 1-15 points was considered as no depression, 16-31 as mild depression, 32-47 as moderate depression, and 48-63 as severe depression.^22^

Then, blood samples were obtained from patients and controls for genetic analysis. Genomic DNA was extracted from blood samples with the QiaAmp DNA Mini Kit (Qiagen, Valencia, CA, USA). To distinguish the L (419 bp) and the S (376 bp) variants of 5-HTTLPR, genotyping was done by polymerase chain reaction (PCR) based on a previously described method.^23^ To detect the A/G single nucleotide polymorphism in the L allele, PCR products were digested with MspI (Vivantis Technologies, Selangor, Malaysia) according to the manufacturer’s recommended procedure.^23^^,^^24^ Genotyping of STin2 VNTR, the 17-bp repeat element in the second intron, was done by PCR to identify alleles STin2.7 (214 bp), STin2.9 (248 bp), STin2.10 (265 bp), and STin2.12 (299 bp).^25^

Allele and haplotype frequencies as well as deviations from the Hardy-Weinberg equilibrium were determined with Arlequin version 3.01 software. Genotypes and genotype combinations were calculated by direct counting. Gene frequencies were compared between patients and controls with the chi-squared or Fisher’s exact tests using Epi Info software (version 6, CDC, Atlanta, GA, USA), and P < 0.05 was considered statistically significant.

## Results

Genetic analysis of the SERT gene was done for 5-HTTLPR, rs35521, and STin2VNTR with PCR-based methods in 100 patients with MS (87 women and 13 men, mean age of onset 31.3 ± 8.5 years) and 100 healthy controls. According to their clinical course, at the time of study 91% of the patients had relapsing-remitting, 8% had secondary progressive, and 1% had primary progressive MS. 

The results of SERT gene analysis are shown in table 1. The frequencies of alleles, genotypes, genotype combinations, and haplotypes did not differ significantly between patients with MS and healthy controls. 

**Table 1:** Serotonin transporter gene polymorphisms in patients with multiple sclerosis (MS) in comparison to healthy control

**Serotonin transporter gene**			**Depression scores (BDI-II) in patients with MS ** **[n (%)]**				**All ** **patients ** **with MS ** **(n = 100)**	**Healthy ** **controls ** **(n = 100)**	**P**
			**Mild ** **depression ** **(n = 26)**	**Moderate ** **depression ** **(n = 15)**	**No ** **depression ** **(n = 52)**	**Undefined ** **(n = 7)**			
Alleles [n (%)]	5-HTTLPR	L_A_	25 (48.1)	12 (40.0)	53 (51.0)	7 (50.0)	97 (48.5)	102 (51.0)	0.34
		L_G_	0 (0)	2 (6.7)	0 (0)	0 (0)	2 (1.0)	0 (0)	
		S	27 (51.9)	16 (53.3)	51 (49.0)	7 (50.0)	101 (50.5)	98 (49.0)	
	STin2VNTR	10	15 (28.8)	10 (33.3)	35 (33.7)	4 (28.6)	64 (32.0)	72 (36.0)	0.39
		12	37 (71.2)	20 (66.7)	69 (66.3)	10 (71.4)	136 (68.0)	128 (64.0)	
Genotypes [n (%)]	5-HTTLPR	L_A_/L_A_	6	3	15	2	26	26	0.67
		L_G_/L_G_	0 (0)	1 (3.3)	0 (0)	0 (0)	1	0	
		L_A_/S	13 (25.0)	6 (20.0)	23 (22.1)	3 (21.4)	45	50	
		S/S	7 (13.5)	5 (16.7)	14 (13.5)	2 (14.3)	28	24	
	STin2VNTR	10/10	0	2	5	1	8	13	0.51
		10/12	15	6	25	2	48	46	
		12/12	11	7	22	4	44	41	
Genotype combinations	L_A_/L_A_, 10/10		0	2	4	1	7	12	0.11
	L_A_/L_A_, 10/12		5	1	10	1	17	10	
	L_A_/L_A_, 12/12		1	0	1	0	2	4	
	L_G_/L_G_, 12/12		0	1	0	0	1	0	
	L_A_/S, 10/10		0	0	0	0	0	1	
	L_A_/S, 10/12		9	5	12	1	27	25	
	L_G_/L_G_, 12/12		4	1	11	2	18	24	
	S/S, 10/10		0	0	1	0	1	0	
	S/S, 10/12		1	0	3	0	4	11	
	S/S, 12/12		6	5	10	2	23	13	
Haplotypes	L_A_-10		0.26	0.33	0.28	0.29	0.28	0.28	NS
	L_A_-12		0.22	0.07	0.23	0.14	0.20	0.23	
	L_G_-12		0.00	0.07	0.00	0.00	0.01	0.00	
	S-10		0.03	0.00	0.06	0.00	0.04	0.08	
	S-12		0.49	0.53	0.43	0.57	0.47	0.41	

MS: Multiple sclerosis; BDI-II: Beck Depression Inventory-II; NS: Not significant

All individuals in the control group were psychologically normal based on their BDI-II score, whereas 26% of patients with MS had mild depression, 15% had moderate depression, 52% were not depressed, and 7% did not complete the inventory correctly. No associations were observed between SERT gene polymorphisms after the patients were stratified for depression status.

Genotype distributions at both loci showed no deviation from the expected Hardy-Weinberg values in patients and controls.

## Discussion

Serotonin is a monoamine neurotransmitter synthesized by tryptophan hydroxylase type 2 (TPH2) in serotonergic neurons of the CNS, where it regulates neurological processes such as anxiety, mood, appetite, sleep, cognition, learning, and memory.^26^ SERT is an integral membrane protein that regulates serotonin levels in the synaptic cleft after neuronal stimulation. It terminates the action of serotonin by rapid reuptake of released serotonin from the synaptic cleft into the presynaptic neuron. Serotonin transporter belongs to the Na^+^/Cl^-^ dependent group of neurotransmitter transporters. Serotonin reuptake into neurons occurs through cotransport with Na^+^ and Cl^-^ and countertransport with K^+^.^27^

We found no remarkable differences in SERT gene polymorphisms between patients with MS and healthy controls. Unlike the control group, 41% of the patients had some degree of depression based on their BDI-II score. Because this questionnaire relies on psychological status during the previous 2 weeks, we are unable to extend these results to other situations. Furthermore, we cannot determine from our results whether depression is a predisposing factor for susceptibility to MS, or is an effect of the disease. MS is a chronic crippling disease which may affect the patient’s whole life. Patients may not be able to do their work, may lose their job, and may have economic problems. Negative expectations about the vague future of their disease and fears that their disease will worsen are likely to contribute to anxiety and depression. 

Although the L variant has been associated with higher levels of SERT gene products and higher reuptake activity, S allele carriers were reported to be more susceptible to depression.^28^^,^^29^ Contrary to the recent report by Saul, et al. who found an association between depression severity in patients with MS with one or two copies of the 5-HTTLPR S allele,^30^ the frequency of S allele carriers in the present study did not differ significantly between patients with MS who reported feeling depressed and those with normal psychological status (53% vs. 51%). Moreover, the frequency of S allele carriers was lower in patients (31%) than in the control group (47%). 

In addition to SERT gene polymorphisms, the modulation of serotonin at the synapses can be regulated by genetic variations in TPH2 and serotonin receptors.^31^ Cofactors such as tetrahydrobiopterin and folic acid are essential for tryptophan hydroxylase activity. This enzyme is also highly sensitive to reactive oxygen species, and chronic inflammation following infection or trauma may damage this enzyme.^32^ Furthermore, natural or chemical serotonin antagonists in foods or dietary supplements can block the effects of serotonin. 

In addition, adequate levels of Na^+^ and Cl^-^ in the extraneuronal space and K^+^ in presynaptic neurons are necessary for optimum SERT functioning,^27^ and altered ion levels in injured neurons may lead to dysregulation of SERT activity in the CNS of patients with MS.^33^

The incidence and prevalence of MS have shown an alarming rise worldwide in recent years.^34^ Although both genetic and environmental factors are thought to be important in the development and exacerbation of MS,^35^ genes are more stable and genetic changes occur slowly, whereas environmental factors can change relatively quickly. The increased prevalence of MS is thus probably related to changes in environmental factors such as infection, diet, air and water pollution, radiation, and stress.^36^ Although studies designed to identify new target genes with genome-wide associations would be helpful, epigenetic studies might be more useful. Epigenetic changes in the interactions with environmental factors may alter the expression pattern of certain genes, which may in turn lead to the induction of MS.^37^

This preliminary study focused on functional polymorphisms of the SERT gene in patients with MS who had different levels of anxiety and depression according to their BDI-II score, in order to shed light on the role of the serotonin-regulating system in mood disorders. This report is the first of its kind, and there are some limitations. To decrease the limitations of the current work, further studies with functional brain imaging of the serotonergic system in patients with MS can better elucidate the role of serotonin in this disease.^27^ This method would clarify the role of SERT in depressed or anxious patients with MS, and provide information of use to determine the optimum pharmacological therapy with antidepressants in these patients. This would be an ultimate goal in these researches at future.^38^

## Conclusion

In addition to SERT gene polymorphisms, modulation of serotonin at the synapses may also be regulated by genetic variations in tryptophan hydroxylase type 2 and serotonin receptors. Further studies with functional brain imaging of the serotonergic system in patients with MS can provide information on the role of serotonin in this disease.

## Notes:

**How to cite this article:** Farjadian Sh, Fakhraei B, Niknam Z, Nasiri M, Azad A, Farjam M, et al. Polymorphisms of serotonin transporter gene and psychological status in patients with multiple sclerosis. Iran J Neurol 2017; 17(3): 105-10.

## References

[1] Noseworthy JH, Lucchinetti C, Rodriguez M, Weinshenker BG (2000). Multiple sclerosis. N Engl J Med.

[2] Farjam M, Zhang GX, Ciric B, Rostami A (2015). Emerging immunopharmacological targets in multiple sclerosis. J Neurol Sci.

[3] Bashinskaya VV, Kulakova OG, Boyko AN, Favorov AV, Favorova OO (2015). A review of genome-wide association studies for multiple sclerosis: Classical and hypothesis-driven approaches. Hum Genet.

[4] Farjam M, Beigi Zarandi FB, Farjadian S, Geramizadeh B, Nikseresht AR, Panjehshahin MR (2014). Inhibition of NR2B-containing N-methyl-D-aspartate receptors (NMDARs) in experimental autoimmune encephalomyelitis, a model of multiple sclerosis. Iran J Pharm Res.

[5] Shi J, Baxter LC, Kuniyoshi SM (2014). Pathologic and imaging correlates of cognitive deficits in multiple sclerosis: Changing the paradigm of diagnosis and prognosis. Cogn Behav Neurol.

[6] Fragoso YD, Adoni T, Anacleto A, da Gama PD, Goncalves MV, Matta AP (2014). Recommendations on diagnosis and treatment of depression in patients with multiple sclerosis. Pract Neurol.

[7] Paparrigopoulos T, Ferentinos P, Kouzoupis A, Koutsis G, Papadimitriou GN (2010). The neuropsychiatry of multiple sclerosis: focus on disorders of mood, affect and behaviour. Int Rev Psychiatry.

[8] Brenner P, Alexanderson K, Bjorkenstam C, Hillert J, Jokinen J, Mittendorfer-Rutz E (2014). Psychiatric diagnoses, medication and risk for disability pension in multiple sclerosis patients; a population-based register study. PLoS One.

[9] Schumann R, Adamaszek M, Sommer N, Kirkby KC (2012). Stress, depression and antidepressant treatment options in patients suffering from multiple sclerosis. Curr Pharm Des.

[10] Araragi N, Lesch KP (2013). Serotonin (5-HT) in the regulation of depression-related emotionality: Insight from 5-HT transporter and tryptophan hydroxylase-2 knockout mouse models. Curr Drug Targets.

[11] Lesch KP, Bengel D, Heils A, Sabol SZ, Greenberg BD, Petri S (1996). Association of anxiety-related traits with a polymorphism in the serotonin transporter gene regulatory region. Science.

[12] Hu XZ, Lipsky RH, Zhu G, Akhtar LA, Taubman J, Greenberg BD (2006). Serotonin transporter promoter gain-of-function genotypes are linked to obsessive-compulsive disorder. Am J Hum Genet.

[13] Lovejoy EA, Scott AC, Fiskerstrand CE, Bubb VJ, Quinn JP (2003). The serotonin transporter intronic VNTR enhancer correlated with a predisposition to affective disorders has distinct regulatory elements within the domain based on the primary DNA sequence of the repeat unit. Eur J Neurosci.

[14] Hofstetter HH, Mossner R, Lesch KP, Linker RA, Toyka KV, Gold R (2005). Absence of reuptake of serotonin influences susceptibility to clinical autoimmune disease and neuroantigen-specific interferon-gamma production in mouse EAE. Clin Exp Immunol.

[15] Hesse S, Moeller F, Petroff D, Lobsien D, Luthardt J, Regenthal R (2014). Erratum to: Altered serotonin transporter availability in patients with multiple sclerosis. European Eur J Nucl Med Mol Imaging.

[16] Hesse S, Moeller F, Petroff D, Lobsien D, Luthardt J, Regenthal R (2014). Altered serotonin transporter availability in patients with multiple sclerosis. Eur J Nucl Med Mol Imaging.

[17] Taler M, Gil-Ad I, Korob I, Weizman A (2011). The immunomodulatory effect of the antidepressant sertraline in an experimental autoimmune encephalomyelitis mouse model of multiple sclerosis. Neuroimmunomodulation.

[18] Mostert JP, Admiraal-Behloul F, Hoogduin JM, Luyendijk J, Heersema DJ, van Buchem MA (2008). Effects of fluoxetine on disease activity in relapsing multiple sclerosis: a double-blind, placebo-controlled, exploratory study. J Neurol Neurosurg Psychiatry.

[19] Mitsonis CI, Zervas IM, Potagas CM, Mitropoulos PA, Dimopoulos NP, Sfagos CA (2010). Effects of escitalopram on stress-related relapses in women with multiple sclerosis: an open-label, randomized, controlled, one-year follow-up study. Eur Neuropsychopharmacol.

[20] Foley P, Lawler A, Chandran S, Mead G (2014). Potential disease-modifying effects of selective serotonin reuptake inhibitors in multiple sclerosis: systematic review and meta-analysis. J Neurol Neurosurg Psychiatry.

[21] Kang H, Metz LM, Traboulsee AL, Eliasziw M, Zhao GJ, Cheng Y (2014). Application and a proposed modification of the 2010 McDonald criteria for the diagnosis of multiple sclerosis in a Canadian cohort of patients with clinically isolated syndromes. Mult Scler.

[22] Ghassemzadeh H, Mojtabai R, Karamghadiri N, Ebrahimkhani N (2005). Psychometric properties of a Persian-language version of the Beck Depression Inventory--Second edition: BDI-II-PERSIAN. Depress Anxiety.

[23] Farjadian S, Fakhraei B, Moeini M, Nasiri M, Fattahi MR (2013). Serotonin transporter gene polymorphisms in Southwestern Iranian patients with irritable bowel syndrome. Arab J Gastroenterol.

[24] Farjadian S, Moghtaderi M, Fakhraei B, Nasiri M, Farjam M (2013). Association between serotonin transporter gene polymorphisms and childhood asthma. J Asthma.

[25] Kaiser R, Muller-Oerlinghausen B, Filler D, Tremblay PB, Berghofer A, Roots I (2002). Correlation between serotonin uptake in human blood platelets with the 44-bp polymorphism and the 17-bp variable number of tandem repeat of the serotonin transporter. Am J Med Genet.

[26] Pytliak M, Vargova V, Mechirova V, Felsoci M (2011). Serotonin receptors - from molecular biology to clinical applications. Physiol Res.

[27] Murphy DL, Lerner A, Rudnick G, Lesch KP (2004). Serotonin transporter: gene, genetic disorders, and pharmacogenetics. Mol Interv.

[28] Nemeroff CB, Owens MJ (2009). The role of serotonin in the pathophysiology of depression: as important as ever. Clin Chem.

[29] Starr LR, Hammen C, Brennan PA, Najman JM (2013). Relational security moderates the effect of serotonin transporter gene polymorphism (5-HTTLPR) on stress generation and depression among adolescents. J Abnorm Child Psychol.

[30] Saul A, Taylor B, Simpson S Jr, Ponsonby AL, Blizzard L, Dwyer T (2018). Polymorphism in the serotonin transporter gene polymorphisms (5-HTTLPR) modifies the association between significant life events and depression in people with multiple sclerosis. Mult Scler.

[31] Miller AL (2008). The methylation, neurotransmitter, and antioxidant connections between folate and depression. Altern Med Rev.

[32] Zhang Q, Raoof M, Chen Y, Sumi Y, Sursal T, Junger W (2010). Circulating mitochondrial DAMPs cause inflammatory responses to injury. Nature.

[33] Friese MA, Schattling B, Fugger L (2014). Mechanisms of neurodegeneration and axonal dysfunction in multiple sclerosis. Nat Rev Neurol.

[34] Kingwell E, Marriott JJ, Jette N, Pringsheim T, Makhani N, Morrow SA (2013). Incidence and prevalence of multiple sclerosis in Europe: A systematic review. BMC Neurol.

[35] Farjam M, Ebrahimpour A, Fakhraei B (2013). CD21 positive B cell: A novel target for treatment of multiple sclerosis. Med Hypotheses.

[36] Vojdani A (2014). A potential link between environmental triggers and autoimmunity. Autoimmune Diseases.

[37] Kucukali CI, Kurtuncu M, Coban A, Cebi M, Tuzun E (2015). Epigenetics of multiple sclerosis: An updated review. Neuromolecular Med.

[38] Hamon M, Blier P (2013). Monoamine neurocircuitry in depression and strategies for new treatments. Prog Neuropsychopharmacol Biol Psychiatry.

